# Molecular epidemiology, genetic diversity, antibiotic resistance and pathogenicity of *Stenotrophomonas maltophilia* complex from bacteremia patients in a tertiary hospital in China for nine years

**DOI:** 10.3389/fmicb.2024.1424241

**Published:** 2024-06-14

**Authors:** Lifeng Wang, Yu Wang, Kun Ye, Xuemei Qiu, Qiang Zhao, LiYan Ye, Jiyong Yang

**Affiliations:** ^1^Department of Laboratory Medicine, First Medical Center of Chinese PLA General Hospital, Beijing, China; ^2^Department of Clinical Laboratory, Shanxi Bethune Hospital, Shanxi Academy of Medical Sciences, Tongji Shanxi Hospital, Third Hospital of Shanxi Medical University, Taiyuan, China

**Keywords:** *Stenotrophomonas maltophilia*, bloodstream infection, whole-genome sequencing, virulence, antibiotic resistance, sequence type

## Abstract

**Background:**

The *Stenotrophomonas maltophilia* complex (Smc) has emerged as a significant nosocomial pathogen contributing to increased mortality rates, particularly in case of bloodstream infections.

**Methods:**

This study employed whole-genome sequencing (WGS) to assess the genetic diversity, antimicrobial resistance profiles, molecular epidemiology and frequencies of virulence genes among 55 *S. maltophilia* isolates obtained from bacteremic cases over a 9-year period.

**Results:**

Based on the threshold of 95% average nucleotide identity (ANI) and 70% digital DNA–DNA hybridization (dDDH) for genospecies delineation, we classified 37 isolates into 6 known species, all belonging to the Smc. The remaining 18 isolates sequenced in this study were assigned to 6 new genomospecies. Among the 55 isolates, we identified 44 different sequence types (STs), comprising 22 known and 22 novel allele combinations. The resistance rate of Smc against trimethoprim-sulfamethoxazole (TMP/SMX) was found to be 3.6%, with the *sul1* and class one integron integrase genes (*intI*) detected in these isolates. All Smc isolates were susceptible to minocycline. Furthermore, all Smc strains harbored the *motA, pilU, smf-1 and Stmpr2* genes. Genomospecies 1 (100%, *n* = 9), *Stenotrophomonas maltophilia* (84.21%, *n* = 19) and *Stenotrophomonas sepilia* (71.43%, *n* = 7) demonstrated a higher percentage of the *afaD* gene, which was also associated with a higher separation rate. In addition to *motA*, *pilU*, *smf-1*, *and Stmpr2* genes, all *S. maltophilia* strains (100%) contained *entA*, *gspD*, *KatA*, and *stmPr1* genes, while all genomospecies 1 strains (100%) contained *afaD, entA*, *gspD*, and *KatA* genes.

**Conclusion:**

Our study highlights the genetic diversity among Smc isolates from patients with bacteremia, revealing 22 novel ST types, 58 new alleles and 6 new genomospecies. *S. maltophilia* and *S. pavanii* were found to carry more virulence factors, emphasizing the importance of accurate strain identification. Minocycline emerged as a promising alternative antibiotic for patients who were resistant to TMP/SMX.

## 1 Introduction

*Stenotrophomonas maltophilia*, previously classified as *Pseudomonas* and later as *Xanthomonas maltophilia*, is a common aerobic non-fermentative Gram-negative bacillus that exists in humid environments, water sources, soil, and plants (Brooke, 2012). The *Stenotrophomonas maltophilia* complex (Smc) has emerged as a significant nosocomial pathogen in clinical settings (Gröschel et al., 2020). Smc is capable of causing a broad spectrum of serious infections, most notably respiratory tract infections and bloodstream infection (BSI). Immunocompromised patients with underlying malignancies, indwelling devices, chronic respiratory diseases, a history of antibiotic use, or prolonged hospital or intensive care unit (ICU) stays are particularly susceptible to severe Smc infections. The mortality rate of patients with Smc bacteremia was 40.5% in a meta-analysis included 19 studies (Huang et al., 2024).

The Smc comprises seven species, including *S. maltophilia*, *S. africana*, *S. pavanii*, *P. hibiscicola*, *P. beteli*, *P. geniculate*, and *S. sepilia* (Ochoa-Sánchez and Vinuesa, 2017; Patil et al., 2018). Due to 16S rRNA gene sequence conservation, taxonomy of Smc is complicated. Taxonogenomics and phylogenomics offer us intra-species and strain-level resolution. Commonly used genome-derived criteria include average nucleotide identity (ANI) and digital DNA–DNA hybridization (dDDH) with species-level cut-offs set at 95 and 70%, respectively (Anzai et al., 2000). More novel genomespecies have been identified in the post-genomic era. The multi-locus sequence typing (MLST) and whole-genome sequencing (WGS) approaches have revealed high genetic heterogeneity among Smc strains. Phylogenetic analyses of 375 nonduplicated Smc genomes have identified at least 20 genogroups (Mercier-Darty et al., 2020).

Due to the intrinsic antimicrobial resistance and acquired resistance to numerous antimicrobial drugs, the treatment of Smc infections can be challenging. Trimethoprim-sulfamethoxazole (TMP/SMX) is the first-line treatment for Smc infections. However, the resistance rate to TMP/SMX has been increasing worldwide. Studies have shown that resistance rates vary according to specimen sources, with Smc isolates from urinary tract infections demonstrating higher resistance to antibiotics compared to isolates from other specimen sources (Vidigal et al., 2014). While Smc possesses a variety of virulence factors, the genes encoding biofilm production and adherence promote their attaching to the surface of medical equipment, which is closely related to BSI (De Oliveira-Garcia et al., 2003; Trifonova and Strateva, 2019). Further study of those virulence factors in Smc causing BSI is warranted.

This study aimed to characterize the genetic diversity, phylogenetic analysis, molecular epidemiology, antimicrobial resistance and virulence genes of Smc isolates obtained from case of bacteremia in China between 2011and 2019 using WGS.

## 2 Materials and methods

### 2.1 Bacterial strains and clinical information

We analyzed 55 nonduplicated clinical strains of Smc were collected between 2011 and 2019 from the Department of Laboratory Medicine, First Medical Center of PLA General Hospital. The isolates were obtained from blood samples and identified as Smc by Matrix-Assisted Laser Desorption/Ionization Time of Flight-Mass Spectrometry (MALDI-TOF MS) (bioMérieux SA, France). Clinical data from the 55 patients, including demographics and respective outcomes, were retrieved from medical records. The diagnosis of bacteremia was made based on both clinical and bacteriological criteria. This study was approved by the Ethics Committee of Chinese PLA General Hospital (No. S2024-349-01). All data were analyzed anonymously.

### 2.2 Susceptibility assays

Minimum inhibitory concentrations (MICs) were determined using the broth microdilution method (Shanghai XingBai Biotechnology Company, Shanghai, China) and interpreted according to the guidelines of the Clinical and Laboratory Standards Institute (CLSI, 2023) interpretive criteria. Seven antibiotics/inhibitors were included in this study: TMP/SMX, ticarcillin/clavulanic acid (TIM), ceftazidime (CAZ), cefepime (FEP), ciprofloxacin (CIP), levofloxacin (LEV), and minocycline (MIN). *Escherichia coli* ATCC 25922 and *Pseudomonas aeruginosa* ATCC 27853 were used as quality control strains for susceptibility testing.

### 2.3 Whole-genome sequencing

Genomic DNA from the strains was extracted using the DNeasy^®^ UltraClean^®^ Microbial Kit (QIAGEN GmbH, 40,724 Hilden, Germany) following the manufacturer’s instructions. The purity and concentration of the DNA were determined using a spectrophotometer. WGS was performed on a HiSeq X Ten sequencer (Illumina Inc., San Diego, CA, United States) using a paired-end library with an average insert size of 350 bp. All sequences were assembled using SOAPdenovo (SOAP Version 2.21). The *N*_50_, *N*_90_, and scaffold number were used to identify *de novo* characteristics (Supplementary Table S2).

### 2.4 Genome similarity assessment and MLST analyses

Genome-based taxonomic methods, including ANI and dDDH, were used for genome similarity assessment, with species-level cut-offs of 95 and 70%, respectively (Kim et al., 2014). *In silico* MLST analysis was performed using the web server of the Centre for Genomic Epidemiology^1^ and then confirmed using the University of Oxford database.^2^ Seven housekeeping genes were used for MLST analysis, including *atpD*, *gapA*, *guaA*, *mutM*, *nuoD*, *ppsA*, and *recA*.

New alleles of the genes were assigned based on the Smc MLST database and the specific sequence type (ST) was determined. When uploading new alleles, the following parameters were used: technology: Illumina; read length: 200–299; coverage: 20-49x; and assembly: *de novo*. The sequences were reversed with Editseq (version: 6.0) to correct star/end sites for the selected locus if necessary. The new alleles were uploaded to the pubMLST database, and the specific STs were then assigned. All genome sequences in this study were submitted to GenBank with BioProject accession number PRJNA675378.

### 2.5 Resistance and virulence genes analysis

The nucleotide sequences of well-characterized antibiotic resistance and virulence genes were retrieved from the complete genome of *S. maltophilia* K279a. The corresponding resistance genes were examined using ResFinder.^3^ The virulence genes encoding biofilm production and adherence were examined using Blast2Seq,^4^ including *afaD*, *entA*, *fliC*, *gspD*, *katA*, *motA*, *pilU*, *rmlA*, *smf-1*, *Stmpr1*, and *Stmpr2.*

### 2.6 Phylogenetic analysis

Single nucleotide polymorphism (SNP) analysis was conducted using the SNP-based CSI Phylogeny 1.4 tool available on the Center for Genomic Epidemiology website^5^ with default parameters for the strains in our study. In total of 59,422 SNPs were found for further analysis. The complete genome sequence of *S. maltophilia* strain K279a (GCF_000072485.1), which was isolated from the blood sample of a patient with bloodstream infection in 1998 (Looney et al., 2009) and generally used as a reference (Gröschel et al., 2020), was also used as a reference in our study. The tree file was visualized using iTOL,^6^ and annotated information was edited using iTOL editor v.1_1.

## 3 Results

### 3.1 Epidemiological data of patients infected with Smc

A total of 55 clinical isolates were obtained from 55 patients with bacteremia at the First Medical Center of Chinese PLA General Hospital between 2011 and 2019. Each isolate was recovered from a single patient. Of these isolates, 40 (72.7%) were from male patients, and 15 (27.3%) were from female patients, with an average age of 48.6 years (range: 4–87 years). The majority of the patients were hospitalized in the Surgical Intensive Care Unit (SICU) (21.8%), followed by the Hematology Department (16.4%), Gastroenterology Department (14.6%) and Hepatobiliary Surgery (14.6%). Among the 55 patients, 12 patients died during hospitalization, resulting in an in-hospital mortality rate of 21.8%.

### 3.2 Genome similarity assessment and discovery of novel genomospecies

To perform genomic classification, ANI and dDDH were calculated (Supplementary Table S1). Based on the threshold of 95% ANI and 70% dDDH for genospecies delineation, 37 isolates were classified into *S. maltophilia, S. pavanii, S. sepilia, P. hibiscicola* and *S. geniculata* and *S. africana*. They were all included in Smc. The remaining 18 isolates sequenced in this study were assigned to 6 novel genomospecies (Genomospecies 1–6). These 6 novel genomospecies displayed ≤93.9% ANI and ≤ 52.1% dDDH compared with the type strains of the genus *Stenotrophomonas*. Additionally, the mortality rates of patients infected with different genomospecies infection varied. The mortality rates of patients infected with *S. maltophilia, S. pavanii, P. hibiscicola, S. sepilia* and genomospecies 2 infected patients were 31.6% (6/19), 50% (3/6), 100% (1/1), 14.3% (1/7), and 25% (1/4) respectively. The demographic and clinical information of the patients were summarized in Supplementary Table S3.

### 3.3 MLST analyses and discovery of new STs

MLST analysis confirmed that the 55 strains of the Smc belonged to 44 different STs. Among these, 22 STs were identified in 30 isolates and have been previously reported in the database. However, 22 STs were reported for the first time in 25 isolates (Table 1). A total of 58 new alleles were identified and assigned new allele numbers by comparison against the pubMLST database. All new allele sequences were deposited in the public databases for molecular typing and microbial genome diversity (pubMLST). The allelic profiles of the strains are shown in Table 1. The most frequently observed ST types were ST-4 (*n* = 3), ST-233 (*n* = 3), and ST-566 (*n* = 3), followed by two strains each belonging to ST-31, ST-24, ST-138, ST-249, and ST-562. The other 36 STs only contained 1 strain.

**Table 1:** The newly discovered MLST profiles of Smc.

Strain number	Alleles							Sequence type
	*atpD*	*gapA*	*guaA*	*mutM*	*nuoD*	*ppsA*	*recA*	
PMA-2	**181**	97	**398**	**222**	**182**	**254**	**213**	**592**
PMA-4	1	1	277	46	7	19	6	**561**
PMA-5	5	4	282	7	25	**247**	**208**	**562**
PMA-13	**177**	**215**	**415**	100	**183**	130	75	**570**
PMA-15	1	96	307	6	6	37	6	**563**
PMA-17	13	172	**399**	50	121	121	22	**564**
PMA-19	14	102	**400**	89	68	**242**	**214**	**565**
PMA-20	103	171	**402**	**217**	72	**251**	166	**577**
PMA-21	74	**217**	**403**	**218**	87	88	**215**	**566**
PMA-23	**179**	**218**	**404**	**219**	12	**250**	13	**594**
PMA-25	74	**217**	**403**	**218**	87	88	**215**	**566**
PMA-26	12	27	352	151	30	29	22	**567**
PMA-31	13	27	**410**	107	30	29	22	**571**
PMA-32	89	167	317	**223**	4	200	158	**572**
PMA-34	74	79	**405**	**218**	87	**249**	**217**	**573**
PMA-35	28	8	218	33	126	200	16	**568**
PMA-37	3	**219**	**406**	105	**186**	108	99	**574**
PMA-39	94	**220**	**407**	119	14	**252**	134	**582**
PMA-42	2	**221**	277	59	2	69	5	**583**
PMA-50	2	**222**	**408**	45	63	168	5	**584**
PMA-51	5	4	282	7	25	**247**	**208**	**562**
PMA-52	13	28	226	151	121	140	22	**569**
PMA-55	13	124	**409**	112	6	144	80	**587**
PMA-56	13	**223**	**411**	83	**184**	**255**	194	**595**
PMA-58	74	**217**	**403**	**218**	87	88	**215**	**566**

In bold: the new ones found in our study.

### 3.4 Phylogenetic analysis

**Figure 1:**
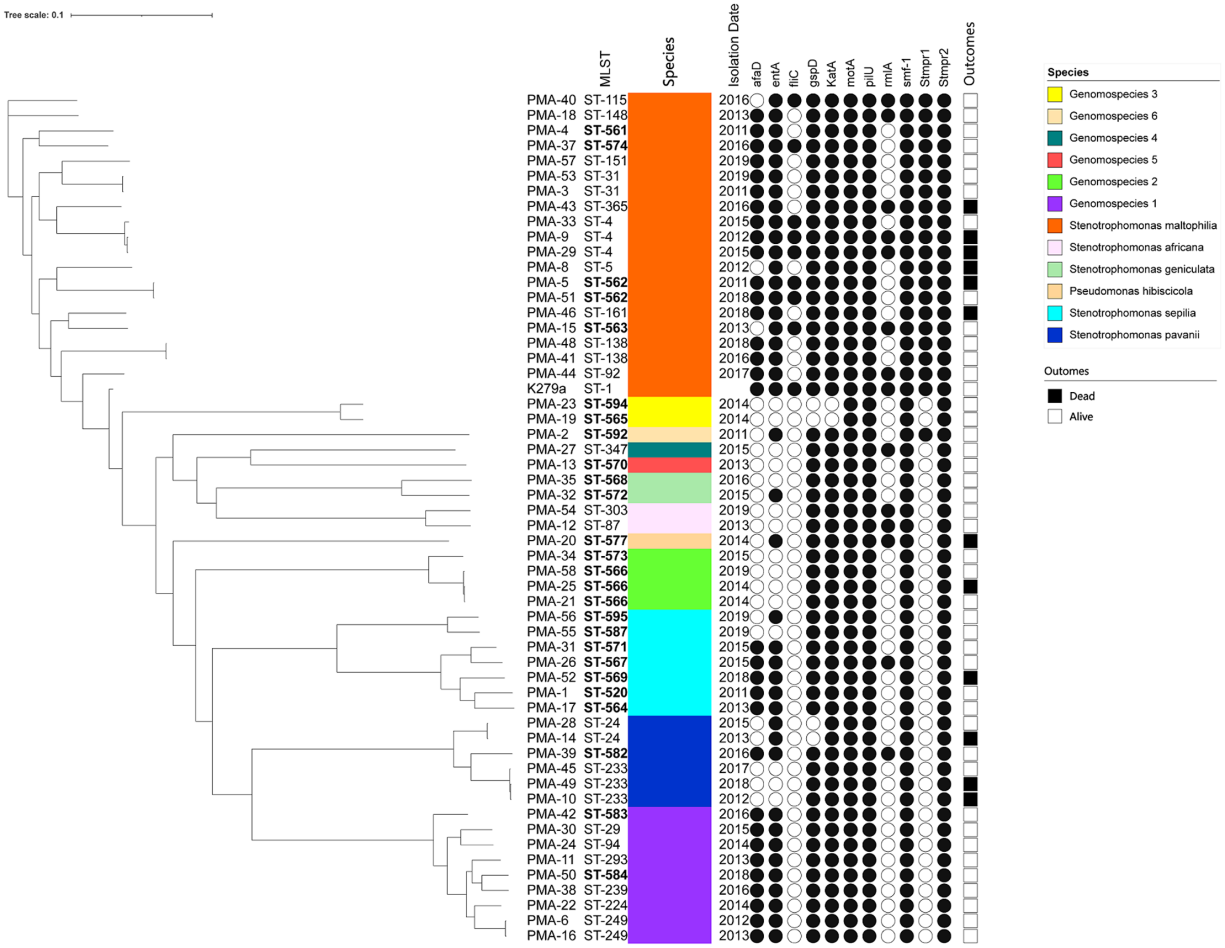
The phylogenic tree of clinical isolates of Smc (*n* = 55). The scale of the tree is shown on the top of the figure and the genetic distance is denoted by branch length. Strains number, MLST types, identification, isolation date, virulence genes and clinical outcomes are listed on the right. The newly discovered ST types are marked in bold. Black and white circles mark the presence and absence of the virulence genes, respectively. Black and white squares on the right side mark the outcomes of died and alive.

A phylogenetic tree based on WGS data is presented in Figure 1. The well-dispersed isolates in the phylogenetic tree suggested no outbreak. However, the tree included a few small clusters, such as ST-4 (*n* = 3), ST-233 (*n* = 3), and ST-566 (*n* = 3). It was noteworthy that two out of the three patients infected with the ST233 strain died.

### 3.5 Antimicrobial susceptibility profile and resistance genes

All Smc strains (100%) were susceptible to MIN. 3.6% (2/55) of the strains (PMA-16 and PMA-43) were resistant to TMP/SMX. Similarly, 3.6% (2/55) of the strains were resistant to LEV (PMA-30 and PMA-43). CAZ resistance was observed in 41.8% (23/55) of the strains. The corresponding MIC_50_, MIC_90_, and the ranges are shown in Table 2. The MICs of each isolate were included in Supplementary Table S4. The sulfonamide-resistance genes (*sul1* and *intI*) were present in two strains (PMA-16 and PMA-43).

**Table 2:** Antibiotic susceptibility of the *S. maltophilia* strains (*n* = 55).

Antibiotics	MIC (μg/mL)			S	I	R
	Range	MIC_50_	MIC_90_			
FEP	4–64	32	64	–	–	–
CIP	0.5–8	2	8	–	–	–
TMP/SMX	19–152	19	19	53 (96.45%)	0	2 (3.6%)
TIM	8–128	16	64	38 (69.1%)	12 (21.8%)	5 (9.1%)
CAZ	4–64	16	64	22 (40.0%)	10 (18.2%)	23 (41.8%)
LEV	1–16	1	2	51 (92.8%)	2 (3.6%)	2 (3.6%)
MIN	2–32	2	2	55 (100.0%)	0	0

S, susceptible; I, intermediate; R, resistant; FEP, cefepime; CIP, ciprofloxacin; TMP/SMX, trimethoprim-sulfamethoxazole; TIM, ticarcillin/clavulanic; CAZ, ceftazidime; MIN, minocycline; LEV, levofloxacin.

### 3.6 Frequency of virulence genes encoding biofilm production and adherence in the Smc strains

The distribution of virulence genes in Smc strains were summarized in Table 3. 100% strains (55/55) harbored the *motA*, *pilU*, *smf-1*, and *Stmpr2* genes. In addition, we analyzed the virulence genes of different genomospecies (Figure 1). *fliC* gene was only detected in *S. maltophilia* (42.11%). In addition to *motA, pilU, smf-1*, and *Stmpr2* genes, 100% *S. maltophilia* strains (19/19) contained *entA*, *gspD*, *KatA*, and *stmPr1* genes, and 100% Genomospecies 1 strains contained *afaD, entA*, *gspD*, and *katA* genes.

**Table 3:** The distribution of virulence genes in Smc strains (%).

Percentage	*afaD*	*entA*	*fliC*	*gspD*	*KatA*	*motA*	*pilU*	*rmlA*	*smf-1*	*Stmpr1*	*Stmpr2*
*S. maltophilia* (*n* = 19)	84.21	100	42.11	100	100	100	100	36.84	100	100	100
*S. sepilia* (*n* = 7)	71.43	85.71	0	100	100	100	100	14.28	100	0	100
*S. pavanii* (*n* = 6)	16.67	50	0	66.67	100	100	100	16.67	100	0	100
*S. geniculata* (*n* = 2)	0	50	0	100	100	100	100	0	100	0	100
*P. hibiscicola* (*n* = 1)	0	100	0	100	100	100	100	100	100	0	100
*S. africana* (*n* = 2)	0	0	0	100	100	100	100	100	100	0	100
Genomospecies 1 (*n* = 9)	100	100	0	100	100	100	100	0	100	0	100
Genomospecies 2 (*n* = 4)	0	0	0	100	100	100	100	0	100	0	100
Genomospecies 3 (*n* = 2)	0	0	0	0	0	100	100	0	100	0	100
Genomospecies 4 (*n* = 1)	0	0	0	100	100	100	100	100	100	0	100
Genomospecies 5 (*n* = 1)	0	0	0	100	100	100	100	0	100	0	100
Genomospecies 6 (*n* = 1)	0	100	0	100	100	100	100	0	100	100	100

## 4 Discussion

Smc has emerged as an important nosocomial pathogen, contributing to increased mortality rates (Trifonova and Strateva, 2019). In our present study, the in-hospital mortality rate of BSIs caused by Smc was 21.8%. WGS was employed to assess the genetic diversity, antimicrobial resistance profiles, molecular epidemiology and frequencies of virulence genes among 55 Smc isolates obtained from bacteremic patients over a 9-year period.

Due to the conservation of the 16S rRNA gene sequence, the taxonomy of Smc is intricate. Phylogenomic and taxonogenomic analyses revealed the heterogeneous structure of Smc. Our study suggested the presence of 6 valid species (*S. maltophilia*, *S. pavanii*, *S. sepilia, P. hibiscicola*, *P. geniculata*, and *S. africana*) within the Smc. Additionally, we identified 6 novel genomospecies. Therefore, the Smc comprised at least 12 distinct genomospecies in our study. Although MALDI-TOF MS is the primary identification method routinely used in most clinical laboratories, accurate identification of Smc should rely on ANI or dDDH. Moreover, the genomes of novel genomospecies could be used as type strain genomes to facilitate accurate species assignments and to aid in the discovery of novel species. Our results indicated that multiple genomospecies of Smc, including potential novel species, were associated with BSIs. *S. maltophilia*, belonging to the core Smc group, was the dominant group among sequenced isolates, followed by genomospecies 1, which represents a putatively novel species. Furthermore, we found that *S. maltophilia* was the most widespread and exhibited a high mortality rate, indicating its status as a high-risk strain. Although the mortality rate associated with *S. pavanii* infection was as high as 50%, further studies are warranted to expand the sample size due to the limited number of strains analyzed in this study.

To date, more than 560 STs of Smc have been deposited in the public databases for molecular typing and microbial genome diversity analysis. Numerous new STs have been reported by different countries (Esposito et al., 2017; Rizek et al., 2018; Bostanghadiri et al., 2019; Mojica et al., 2019). In our present study, we identified a total of 22 novel ST types and 58 new alleles among the isolates. Our results demonstrated a striking diversity of Smc genotype with the STs well dispersed, consistent with previous findings (Mojica et al., 2019; Yinsai et al., 2023). Genome dynamics play a crucial role in the survival and evolution of bacteria. Smc illustrates the evolutionary intricacies of an opportunistic pathogen with the emergence of novel STs posing a potential major threat in healthcare settings. While Smc outbreaks are commonly reported from respiratory specimens, BSI outbreaks are rare. In our 9-year study period, no outbreak was observed.

Smc is the third most common nosocomial non-fermenting Gram-negative bacillus and exhibits high levels of intrinsic and acquired resistance to multi-antibacterial agents (Brooke, 2014). In recent years, multidrug-resistant Smc isolates have become increasingly prevalent in certain regions, posing significant challenges to treatment (Rizek et al., 2018; Kumar et al., 2020). In our study, the resistance rate of Smc against TMP/SMX was 3.6%, with the *sul1* and *intI* detected in these isolates, mediating TMP/SMX resistance (Hu et al., 2011). However, some studies have reported higher rates of TMP/SMX resistance, ranging from 13.7 to 32.8%, with 61.3 to 100% of Smc isolates originating from the respiratory tract (Flores-Treviño et al., 2014; Adegoke et al., 2017). These discrepancies may be attributed to differences in specimen types. All Smc isolates in our study were susceptible to MIN, suggesting its potential use as a therapeutic alternative. However, Smc exhibited a high resistance rate against CAZ, reaching up to 41.8%. Previous studies have suggested that low membrane permeability, the presence of drug efflux pumps and variations in the efflux pump genes may induce resistance expect drug hydrolyzing genes (Crossman et al., 2008).

Furthermore, we analyzed the presence of virulence genes among different genomospecies of Smc. All Smc strains harbored *motA, pilU, smf-1 and Stmpr2* genes. Interestingly, the *fliC* (flagellin) gene was only detected in *S. maltophilia* with a prevalence of 42.11%. A previous study has reported that all *S. maltophilia* strains that originated from blood samples show 100% (17/17) positivity for *fliC* gene, it is worth noting that there was clonal transmission among these strains (Cruz-Córdova et al., 2020). Strains with a higher percentage of *afaD* gene included genomospecies 1 (100%, *n* = 9), *S. maltophilia* (84.21%, *n* = 19) and *S. sepilia* (71.43%, *n* = 7), which were also the strains with higher rates of separation. Smc strains isolated from blood samples showed a high percentage of the *fliC* and *afaD* genes which are associated with colonization (Cruz-Córdova et al., 2020). The finding suggested that these virulence factors might contribute to the persistence and dissemination of Smc strains. The *stmPr1* gene encoding extracellular protease only found in *S. maltophilia* and genomospecies 6, indicating its potential utility for the preliminary identification of strains. In addition to *motA, pilU, smf-1*, and *Stmpr2* genes, 100% of *S. maltophilia* strains also contained the *entA*, *gspD*, *katA*, and *stmPr1* genes, and 100% of genomospecies 1 strains contained the *afaD, entA*, *gspD*, and *katA* genes. The presence of varied virulence factors among different genomospecies underscored the importance of accurate strain identification. There are many factors affecting mortality rate, especially the immune status and underlying diseases of the patients, therefore we did not observe a correlation between virulence genes (encoding biofilm production and adherence) and mortality.

In conclusion, our study highlights the genetic diversity among Smc isolates from bacteremic patients and revealed 22 novel ST types, 58 new alleles and 6 new genomospecies. Our findings indicated that at least 12 distinct genomospecies were associated with BSIs in our hospital. MIN emerged as a potential alternative antibiotic for patients who were resistant to TMP/SMX. Both *S. maltophilia* and *S. pavanii* harbored a higher number of virulence factors and were associated with high mortality rates. Therefore, accurate strain identification is very important. Our data suggested no outbreak at our testing center. However, the limited number of collected strains in this single-center study might restricted data analysis to identify transmission relationships within the local area. Therefore, a large-scale, multicenter study should be carried out in the future.

## Data availability statement

The datasets presented in this study can be found in online repositories. The names of the repository/repositories and accession number(s) can be found in the article/Supplementary material.

## Author contributions

LW: Conceptualization, Investigation, Validation, Writing – original draft, Writing – review & editing. YW: Investigation, Writing – original draft. KY: Data curation, Methodology, Writing – original draft. XQ: Validation, Visualization, Writing – review & editing. QZ: Methodology, Writing – review & editing. LY: Formal analysis, Writing – review & editing. JY: Conceptualization, Supervision, Writing – review & editing.

## Footnotes

^1^https://cge.cbs.dtu.dk/services/MLST/

^2^https://pubmlst.org/smaltophilia/

^3^https://cge.cbs.dtu.dk/services/ResFinder/

^4^https://blast.ncbi.nlm.nih.gov/Blast.cgi

^5^https://cge.cbs.dtu.dk/services/CSIPhylogeny/

^6^https://itol.embl.de
