# Supplementary material for: Molecular epidemiology, genetic diversity, antibiotic resistance and pathogenicity of Stenotrophomonas maltophilia complex from bacteremia patients in a tertiary hospital in China for nine years
Source: Front Microbiol. 2024 Jun 14;15:1424241. doi: 10.3389/fmicb.2024.1424241 (PMC11211261; doi:10.3389/fmicb.2024.1424241)
Supplement: Supplementary file 1 [file Data_Sheet_1.pdf]

Supplementary Table 1 ANI and dDDH values among six novel genomospecies with the type strains of the genus *Stenotrophomonas*

Reference strain	**Sm42**		**Sm21**		**sm23**		**Sm27**		**sm13**		**Sm2**	
	ANI	dDDH	ANI	dDDH	ANI	dDDH	ANI	dDDH	ANI	dDDH	ANI	dDDH
Pseudomonas hibiscicola ATCC_19867	92.5	45.7	91.7	43.1	88.2	33.5	92.5	46.2	92.2	44.2	92.1	44.4
Stenotrophomonas acidaminiphila	83.9	23.9	83.8	23.9	83.1	23.2	83.7	23.9	83.6	23.6	83.7	23.8
Stenotrophomonas bentonitica	85.0	25.3	85.0	24.8	84.1	24.1	85.0	25.1	84.8	24.8	84.9	25.0
Stenotrophomonas chelatiphaga	84.9	25.7	84.7	25.5	84.1	24.8	84.7	25.6	84.6	25.5	84.8	25.6
Stenotrophomonas cyclobalanopsidis	89.0	34.1	88.9	34.0	87.6	31.8	88.9	34.0	89.0	33.9	88.9	34.2
Stenotrophomonas daejeonensis	83.8	23.9	83.6	23.7	83.1	23.4	83.7	23.8	83.5	24.0	83.5	24.0
Stenotrophomonas geniculate N1	92.2	44.4	92.0	43.8	88.3	33.6	93.6	50.4	93.0	47.9	92.8	46.8
Stenotrophomonas ginsengisoli	81.7	21.2	81.5	21.0	81.1	20.9	81.5	21.1	81.4	21.3	81.4	21.3
Stenotrophomonas humi	82.3	22.8	82.2	22.4	81.7	22.3	82.4	22.4	82.1	22.5	82.3	22.6
Stenotrophomonas indicatrix	88.6	32.4	88.4	32.3	87.4	31.4	88.5	32.5	88.5	32.3	88.4	32.2
Stenotrophomonas koreensis	81.3	21.2	81.3	21.0	81.1	20.9	81.2	20.8	81.2	21.1	81.3	20.9
Stenotrophomonas lactitubi	88.5	32.5	88.4	32.4	87.2	31.3	88.4	32.6	88.5	32.3	88.5	32.1
Stenotrophomonas maltophilia	92.5	44.9	91.8	43.2	88.2	33.7	93.0	48.5	92.6	46.1	93.9	52.1
Stenotrophomonas nematodicola	84.5	24.4	84.6	24.0	83.9	23.6	84.7	24.8	84.4	24.2	84.5	24.2
Stenotrophomonas nitritireducens	83.9	24.0	83.8	24.0	83.2	23.4	83.9	24.0	83.7	24.0	83.7	23.8
Stenotrophomonas panacihumi	82.9	22.4	82.8	22.2	82.3	21.6	82.8	22.3	82.7	22.3	82.9	22.4
Stenotrophomonas pavanii	93.7	51.5	92.2	45.5	88.5	34.0	91.4	42.6	91.3	42.1	91.3	42.0
Stenotrophomonas pennii	84.7	25.2	84.6	25.1	84.0	24.4	84.6	25.3	84.5	25.3	84.6	25.3
Stenotrophomonas pictorum JCM9942	82.9	23.7	82.8	23.5	82.3	23.2	82.9	23.9	82.8	23.7	82.8	23.9
Stenotrophomonas rhizophila	84.9	24.7	84.8	24.7	83.9	24.0	84.8	24.7	84.8	24.6	84.9	24.6
Stenotrophomonas sepilia	93.2	49.0	92.5	46.3	88.2	33.5	91.8	44.2	91.7	43.3	91.5	42.7
Stenotrophomonas terrae	82.6	22.7	82.3	22.7	81.8	22.4	82.6	22.6	82.3	22.8	82.5	22.8
Stenotrophomonas tumulicola	84.2	25.3	84.1	25.1	83.6	24.4	84.0	25.2	84.5	25.9	84.1	25.3
Stenotrophomonas africana	92.4	45.3	91.8	43.6	88.2	33.4	92.8	47.8	93.5	50.7	92.5	46.2

Supplementary Table 2 The assembling / sequencing characteristics of 55 genomes

strains	Total length (bp)	scaffold number	N50 (bp)	N90 (bp)
PMA-1	4422041	57	422092	64856
PMA-2	4701517	43	976763	261186
PMA-3	4638013	95	100374	40951
PMA-4	4595265	104	150949	43136
PMA-5	4533239	58	436629	176435
PMA-6	4603591	53	278580	130422
PMA-8	4752494	59	661550	84604
PMA-9	4607096	86	218353	52259
PMA-10	4686181	140	116425	33654
PMA-11	4482817	74	416344	91562
PMA-12	4903518	73	486664	103018
PMA-13	4907009	50	1126255	222661
PMA-14	4610897	101	271524	87398
PMA-15	4573592	89	269455	64142
PMA-16	4684247	55	436406	238911

PMA-17	4418485	49	281373	102369
PMA-18	4623196	95	158177	60693
PMA-19	4119993	48	390946	121782
PMA-20	4304884	69	253816	86235
PMA-21	4533338	47	442392	182738
PMA-22	4804338	80	151903	68219
PMA-23	4116152	51	456243	196358
PMA-24	4806273	73	379771	96522
PMA-25	4533441	48	570989	226936
PMA-26	4585317	58	174798	63668
PMA-27	4986683	122	122148	33414
PMA-28	4665740	203	30144	9369
PMA-29	4790743	104	166084	40959
PMA-30	4548501	93	224221	47952
PMA-31	4433203	76	235832	51924
PMA-32	4544904	46	533064	140725
PMA-33	4770213	90	202322	95156
PMA-34	4553212	95	281579	93068
PMA-35	4521758	38	380759	90117
PMA-37	4491407	98	161727	48924
PMA-38	4885410	76	302292	83352
PMA-39	4452984	69	491337	107713
PMA-40	4613033	88	182274	53622
PMA-41	4715316	76	153504	55096
PMA-42	4490315	93	244791	66692
PMA-43	4655199	96	190683	49132
PMA-44	4859130	128	251318	47373
PMA-45	4665318	114	124439	38196
PMA-46	4609278	92	125292	37554
PMA-48	4631478	844	31020	9210
PMA-49	4693048	108	235149	73771
PMA-50	4503926	81	288814	122524
PMA-51	4556049	57	816597	147277
PMA-52	4362440	114	155267	49832
PMA-53	4690961	101	120081	42307
PMA-54	4991160	67	177044	82749
PMA-55	4411064	84	258783	52618
PMA-56	4857598	75	165813	48707
PMA-57	4814217	100	174887	43827
PMA-58	4694767	54	392815	166093

Supplementary Table 3 The demographic and clinical information of the 55 patients

Patients	Genomospecies	Gender	Aged	Underlying Diseases	Hospital stay (days)	Treatment	outcomes
PMA-1	*S. sepilia*	Female	54	Acute monocytic leukemia	61	Levofloxacin	Alive
PMA-2	Genomospecies 6	Female	47	Hypertension	10	Levofloxacin	Alive
PMA-3	*S. maltophilia*	Male	77	Diabetes; Cerebral infarction	23	Cefmetazole	Alive
PMA-4	*S. maltophilia*	Male	39	Cerebral infarction	49	Ceftriaxone	Alive
PMA-5	*S. maltophilia*	Male	77	Lung cancer	27	Imipenem	Died
PMA-6	Genomospecies 1	Male	74	Cholangiocarcinoma; Hypertension	32	Levofloxacin	Alive
PMA-8	*S. maltophilia*	Male	38	Abdominal infection	18	Tegecycline	Died
PMA-9	*S. maltophilia*	Male	4	Neuroblastoma	29	Ceftriaxone	Died
PMA-10	*S. pavanii*	Male	27	Acute lymphocytic leukemia	40	Cefoperazone sulbactam	Died
PMA-11	Genomospecies 1	Female	23	Acute lymphocytic leukemia	32	Ceftazidime tazobactam	Alive
PMA-12	*S. africana*	Male	63	Obstructive jaundice; Cholangitis	25	Trimethoprim-sulfamethoxazole	Alive
PMA-13	Genomospecies 5	Male	34	Aconitum alkaloid poisoning	37	Piperacillin tazobactam	Alive
PMA-14	*S. pavanii*	Male	21	Acute myeloid leukemia	42	Imipenem	Died
PMA-15	*S. maltophilia*	Male	52	Cardiovascular disease	9	Piperacillin tazobactam	Alive
PMA-16	Genomospecies 1	Male	34	Cholangitis	11	Biapenem	Alive
PMA-17	*S. sepilia*	Female	48	Uremia	13	Piperacillin tazobactam	Alive
PMA-18	*S. maltophilia*	Male	37	Acute pancreatitis	18	Tegecycline and Cefoperazone sulbactam	Alive
PMA-19	Genomospecies 3	Male	73	Obstructive jaundice; Hypertension	21	Cefoperazone sulbactam	Alive
PMA-20	*P. hibiscicola*	Male	84	Cholangitis	20	Imipenem	Died
PMA-21	Genomospecies 2	Male	85	Diabetes; Hypertension	153	Piperacillin tazobactam	Alive
PMA-22	Genomospecies 1	Female	72	Cholangiocarcinoma; Hypertension	50	Cefoperazone sulbactam	Alive
PMA-23	Genomospecies 3	Female	66	Gallbladder cancer	21	Trimethoprim-sulfamethoxazole	Alive
PMA-24	Genomospecies 1	Female	37	Acute lymphocytic leukemia	54	Ceftriaxone	Alive
PMA-25	Genomospecies 2	Male	52	Hydronephrosis	106	Ceftazidime tazobactam	Died
PMA-26	*S. sepilia*	Male	23	Acute myeloid leukemia	74	Trimethoprim-sulfamethoxazole	Alive
PMA-27	Genomospecies 4	Male	74	Gastric cancer; Diabetes; Hypertension	12	Cefoperazone sulbactam	Alive
PMA-28	*S. pavanii*	Male	34	Acute pancreatitis	75	Cefoperazone sulbactam	Alive
PMA-29	*S. maltophilia*	Male	66	Pancreatic cancer; Diabetes; Hypertension	58	Minocycline	Died
PMA-30	Genomospecies 1	Male	69	Acute myeloid leukemia	48	Cefoperazone sulbactam	Alive
PMA-31	*S. sepilia*	Female	52	Acute myeloid leukemia	23	Levofloxacin	Alive
PMA-32	*S. geniculata*	Male	70	Rectal cancer	96	Cefoperazone sulbactam	Alive
PMA-33	*S. maltophilia*	Female	56	Acute pancreatitis	117	Cefoperazone sulbactam and	Alive

						Moxifloxacin	
PMA-34	Genomospecies 2	Male	52	Trauma	26	Cefoperazone sulbactam	Alive
PMA-35	*S. geniculata*	Male	64	Obstructive jaundice; Cholangitis; Diabetes	31	Cefoperazone sulbactam	Alive
PMA-37	*S. maltophilia*	Female	32	Systemic lupus erythematosus; Endocarditis	76	Cefoperazone sulbactam	Alive
PMA-38	Genomospecies 1	Male	58	Mitral Valve Replacement	55	Cefoperazone sulbactam	Alive
PMA-39	*S. pavanii*	Male	64	Tongue cancer	96	Levofloxacin	Alive
PMA-40	*S. maltophilia*	Male	79	Acute intestinal obstruction	56	Cefoperazone sulbactam	Alive
PMA-41	*S. maltophilia*	Male	39	Acute lymphocytic leukemia	1	Moxifloxacin	Alive
PMA-42	Genomospecies 1	Male	57	Chronic Kidney Disease; Hypertension	31	Ceftriaxone	Alive
PMA-43	*S. maltophilia*	Female	22	Trauma	73	Cefoperazone sulbactam	Died
PMA-44	*S. maltophilia*	Female	60	Acute lymphocytic leukemia	40	Cefoperazone sulbactam	Alive
PMA-45	*S. pavanii*	Female	65	Acute intestinal obstruction	27	Levofloxacin	Alive
PMA-46	*S. maltophilia*	Female	87	Cholangiocarcinoma	27	Levofloxacin	Died
PMA-48	*S. maltophilia*	Male	42	Acute cholangitis	31	Meropenem	Alive
PMA-49	*S. pavanii*	Male	21	Aplastic anemia	136	aztreonam	Died
PMA-50	Genomospecies 1	Male	58	Liver cancer	17	Tegecycline	Alive
PMA-51	*S. maltophilia*	Male	33	Cholangitis	0	——	——
PMA-52	*S. sepilia*	Male	56	Liver cancer	74	Levofloxacin	Died
PMA-53	*S. maltophilia*	Male	69	Abdominal tumor	11	Cefoperazone sulbactam and Trimethoprim-sulfamethoxazole	Alive
PMA-54	*S. africana*	Male	48	Chronic Kidney Disease; Diabetes	16	Biapenem	Alive
PMA-55	*S. sepilia*	Male	32	Acute myeloid leukemia	28	Trimethoprim-sulfamethoxazole	Alive
PMA-56	*S. sepilia*	Male	61	Esophageal squamous cell carcinomas	87	Levofloxacin	Alive
PMA-57	*S. maltophilia*	Female	69	Gastric cancer	29	Tegecycline	Alive
PMA-58	Genomospecies 2	Male	62	Severe pancreatitis	48	Tegecycline	Alive

The patient (PMA-51) was an outpatient with unknown treatment and prognosis.

Supplementary Table 4. MIC of the 55 Smc strains

Strains	MIC (μg/mL)						
	FEP	CIP	TMP/SMX	TIM	CAZ	LEV	MIN
PMA-1	64	4	1/19	64/2	8	2	2
PMA-2	32	2	1/19	8/2	16	1	2
PMA-3	32	1	1/19	8/2	32	1	2
PMA-4	16	2	1/19	8/2	16	1	2
PMA-5	64	1	2/38	8/2	32	1	2
PMA-6	16	1	1/19	16/2	4	1	2
PMA-8	32	2	1/19	8/2	32	1	2
PMA-9	>64	4	1/19	128/2	>64	2	2
PMA-10	32	8	1/19	8/2	16	2	2
PMA-11	32	1	1/19	8/2	4	1	2
PMA-12	16	8	1/19	>128/2	32	2	4
PMA-13	32	1	1/19	32/2	32	1	2
PMA-14	32	1	1/19	8/2	4	1	2
PMA-15	64	2	1/19	8/2	32	1	2
PMA-16	32	8	8/152	64/2	4	4	2
PMA-17	32	2	1/19	64/2	8	1	2
PMA-18	64	2	1/19	8/2	64	2	2
PMA-19	64	2	1/19	8/2	16	1	2
PMA-20	32	2	1/19	32/2	16	1	2
PMA-21	32	2	1/19	8/2	4	1	2
PMA-22	8	1	1/19	8/2	4	1	2
PMA-23	64	2	2/38	16/2	16	1	2
PMA-24	4	4	1/19	8/2	4	2	2
PMA-25	32	2	1/19	16/2	4	1	2
PMA-26	64	2	1/19	8/2	4	1	2
PMA-27	32	2	1/19	8/2	64	1	2
PMA-28	32	2	1/19	16/2	16	1	2
PMA-29	32	4	1/19	16/2	32	2	2
PMA-30	32	8	1/19	64/2	8	8	2
PMA-31	8	1	1/19	8/2	4	1	2
PMA-32	64	2	1/19	>128/2	>64	1	2
PMA-33	16	1	1/19	8/2	16	1	2
PMA-34	16	2	1/19	32/2	4	2	2
PMA-35	>64	2	1/19	>128/2	>64	1	2
PMA-37	64	2	1/19	64/2	>64	1	2
PMA-38	32	1	1/19	8/2	4	1	2
PMA-39	32	1	1/19	8/2	4	1	2
PMA-40	16	2	1/19	16/2	32	1	2
PMA-41	16	2	1/19	8/2	8	1	2
PMA-42	32	2	1/19	8/2	4	1	2
PMA-43	32	>8	8/152	8/2	16	8	2
PMA-44	>64	4	1/19	16/2	64	4	2
PMA-45	32	4	1/19	8/2	4	1	2
PMA-46	64	2	1/19	16/2	64	1	2
PMA-48	32	2	1/19	16/2	32	1	2
PMA-49	64	4	1/19	64/2	32	1	2
PMA-50	64	2	1/19	64/2	8	2	2
PMA-51	>64	1	1/19	>128/2	>64	1	2
PMA-52	64	1	1/19	32/2	4	1	2
PMA-53	64	2	1/19	16/2	32	2	2
PMA-54	64	0.5	1/19	32/2	32	1	2
PMA-55	64	2	1/19	8/2	16	1	2
PMA-56	64	4	1/19	8/2	32	2	2
PMA-57	32	2	1/19	16/2	64	1	2
PMA-58	32	2	1/19	8/2	4	1	2
